# Supplementary figures and images for: Can we detect conditioned variation in political speech? two kinds of discussion and types of conversation
Source: PLoS One. 2021 Feb 11;16(2):e0246689. doi: 10.1371/journal.pone.0246689 (PMC7877629; doi:10.1371/journal.pone.0246689)

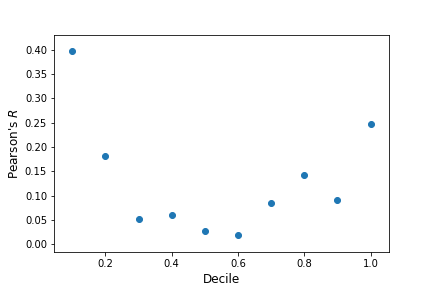

Supplement: S1 Fig — The x-axis indicates deciles of the distribution of logoddsR values (calculated using the Congressional Record) of the 2,408 words that appear in both the Congressional Record and presidential debates corpora. Words in the leftmost bins are highly Democratic (have a very low corresponding logoddsR), while words in the rightmost bins are highly Republican (have a very high corresponding logoddsR). On the y-axis are the Pearson’s correlation coefficients between the logoddsR values calculated using the Congressional Record and the presidential debates for the words that fall in each bin. For example, when the sample is restricted to the 241 words whose corresponding logoddsR was above the 10% and below the 20% cut points, the correlation is.18. The U-shape indicates that this correlation is highest for words with higher absolute values of logoddsR. The highest correlation (.40) occurs for words in the 10% decile (the most Democratic words). The lowest correlation (.02) occurs for words in the 60% decile. (PNG) [file pone.0246689.s001.png]

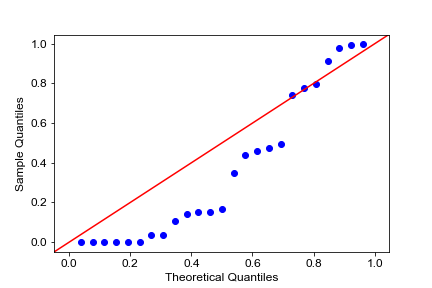

Supplement: S2 Fig — The closer the points fall to the red diagonal line, the more the distribution of p-values resembles what would be expected under the null hypothesis. The observed pattern shows that most p-values are smaller than would be expected under the null hypothesis, although the highest p-values, which correspond to the items that show a significant effect in the opposite direction of our hypothesis (see caption for S1 File), are higher than would be expected under the null. (PNG) [file pone.0246689.s002.png]
