# Supplementary material for: Can we detect conditioned variation in political speech? two kinds of discussion and types of conversation
Source: PLoS One. 2021 Feb 11;16(2):e0246689. doi: 10.1371/journal.pone.0246689 (PMC7877629; doi:10.1371/journal.pone.0246689)
Supplement: S1 Appendix — (PDF) [file pone.0246689.s004.pdf]

A Exclusion criteria for words

When pre-processing the natural language data from the U.S. Congressional Record and presidential debates, we excluded all words that are in one or more of the following sets:

- digits,
- common stopwords (e.g. pronouns such as "i" and "you", common and functionally versatile verbs such as "be" and "have," and prepositions such as "about" and "during;" following Gentzkow et al. (2019) [1], we use the list of stopwords made available at http://snowball.tartarus.org/algorithms/english/stop.txt),
- "US-Congress-specific" stopwords taken from Table 9 of Gentzkow et al.(2019) [1] (e.g. "adjourn," "chairman" and "yield"),
- names of states (accessed from liststates.com),
- names of the federal districts and inhabited territories (accessed from https://en.wikipedia.org/wiki/List_of_states_and_territories_of_the_United_States [2]),
- the names of countries (accessed from https://www.state.gov/misc/list/index.htm),

- the name of major U.S. cities (accessed from https://www.biggestuscities.com/),
- the last names of all Congressmen and senators who sat from 2012–2017 (accessed from https://github.com/unitedstates/congress-legislators),
- the names of all presidential candidates in the 2012 and 2016 election cycles (accessed from The American Presidency Project [3]),
- words that Democrats and Republicans each said less than 10 times, and
- procedural words. We use the following criteria to identify procedural words:
  - the word is contained in a bigram that's said in more than 1% of speeches, AND
  - the word is contained in a bigram that's said more than 10 times as frequently as in general speech (data on the frequency of words in general speech is obtained from the Corpus of Contemporary American English (COCA) corpus [4], AND
  - in the majority of contexts in which the word is spoken, it is preceded and followed by the same words (determined by manual coding of three random excerpts in which the word appeared).

References

1. Gentzkow M, Shapiro JM, Taddy M. Measuring Group Differences in High-Dimensional Choices: Method and Application to Congressional Speech. Econometrica. 2019;87(4):1307–1340. doi:10.3982/ECTA16566.
2. List of States and Territories of the United States;. https://en.wikipedia.org/wiki/List_of_states_and_territories_of_the_United_States.
3. UC Santa Barbara. The American Presidency Project;. http://www.presidency.ucsb.edu/debates.php.
4. Davies M. N-Grams Data from the Corpus of Contemporary American English (COCA); 2011. http://www.ngrams.info.
