# Supplementary material for: Can we detect conditioned variation in political speech? two kinds of discussion and types of conversation
Source: PLoS One. 2021 Feb 11;16(2):e0246689. doi: 10.1371/journal.pone.0246689 (PMC7877629; doi:10.1371/journal.pone.0246689)
Supplement: S2 Appendix — (PDF) [file pone.0246689.s005.pdf]

B Study materials

B.1 Study 1 materials

B.1.1 Task instructions

Participants were presented with 100 words in a random order: 78 words selected on the basis of partisanship (described in our discussion of Study 1), 20 words included to test the social valence bias hypothesis (reported in Sloman et al. (under review) [1]), and two words randomly reselected from the other 98 as attention check questions. The order of the words "Democrat" and "Republican" in the instructions was randomized.

> On each of the next five pages, you will see a list of 20 words. For each word, imagine you were watching a congressional debate on C-Span and you

heard that word. Please estimate how likely it is that the word is spoken either by a Democrat or by a Republican [Republican or by a Democrat].

Your responses will be most helpful if you go with your first impression, so we ask that you spend no more than 5 seconds on each word—but feel free to take breaks in between pages.

B.1.2 Stimuli

Democratic words		Republican words	
bill	cut	president	move
women	communities	obamacare	irs
republicans	pay	government	look
health	food	law	world
public	billion	obama*	folks
million	deficit	going	within
cuts	benefits	regulations	appreciate
care	water	god	power
americans	climate	energy	businesses
gun	transportation	got	days
families	trump*	life	extend
affordable*	american	constitution	article
congress	millions	spending	service
children	poverty	freedom	regulatory
violence	safety	small	states
need	students	years	year
country	protections	actually	little
education	immigration	business	bureaucrats
class	income	things	radical
people		federal	

* Excluded from analysis after being designated as "procedural words."

Valenced words (not included in reported analyses)

Positive words	Negative words
joy	sorrow
superior	inferior
plentiful	scarce
qualified	unqualified
laugh	cry
famous	unknown
clever	stupid
praise	blame
sweet	bitter
accurate	inaccurate

B.2 Study 2 materials

B.2.1 Task instructions

Participants were presented with 100 word pairs in a random order: 88 word pairs selected on the basis of partisanship (described in our discussion of Study 2), 10 word pairs included to test the social valence bias hypothesis (reported in Sloman et al. (under review) [1]), and two pairs randomly reselected from the other 98 as attention check questions. Each participant was randomly assigned to select whether each word was indicative that the speaker was a Democrat or a Republican.

> If you have ever watched C-Span, you may have felt like sometimes you can guess a person's party by the words that they use. The next few pages contain 100 word pairs, most of which are sampled from speeches in the United States Congress. Most pairs contain a word indicative that the speaker is a Democrat and a word indicative that the speaker is a Republican. For each word pair, please guess which is indicative that the speaker is a DEMOCRAT [REPUBLICAN].
>
> Your responses will be most helpful if you go with your first impression, so we ask that you spend no more than 5 seconds on each word pair—but feel free to take breaks in between pages!

B.2.2 Stimuli

Democratic word	Republican word	Democratic word	Republican word
colleague	friend	capacity	capability
particularly	especially	deny	give
acknowledge	recognize	brought	bringing
measure	legislation	provide	deliver
months	years	supported	sponsored
essentially	basically	protect	defend
indicated	stated	country	globe
extraordinary	incredible	democracy	freedom
mother	father	ensure	ensures
sensible	commonsense	protecting	defending
billion	trillion	rates	prices
immigrants	aliens	program	project
view	opinion	consumers	taxpayers
want*	wanted*	children	babies
moreover	additionally	policy	policies
aca	obamacare	create	produce
evening	morning	institute	foundation
people	folks	deficit	debt
week	year	rape	murder
pleased	happy	leaving	left
seconds	minutes	difficult	tough
companies	manufacturers	statement	comments
changes	reforms	nothing	something
able	willing	sexual	sex
values	principles	homeowners	farmers
underserved	rural	investors	inventors
problems	problem	commitments	promises
adopted	passed	wars	war
bad	good	require	include
cities	counties	question	questions
woman	man	programs	activities
undermine	destroy	ports	borders
eliminates	provides	bridge	fence
undocumented	illegal	partners	allies
cuts	increases	corporations	businesses
tax	taxes	agreement	treaty
workers	employees	hope	believe
parks	forests	shutdown	takeover
congress	body	police	fire
oppose	support	americans	hoosiers
lives	life	delta	lake
concerned	talking	region	area
colleagues	friends	taking	takes
seniors	patients	enable	encourage

* Excluded from analysis after pre-processing using later versions of the list of stopwords led to *want* reversing polarity.

Valenced words (not included in reported analyses)

Positive words	Negative words
superior	inferior
joy	sorrow
plentiful	scarce
qualified	unqualified
laugh	cry
clever	stupid
rapid	slow
famous	unknown
useful	useless
loyal	disloyal

B.3 Study 3a materials

B.3.1 Task instructions

The order of the words "Democrat" and "Republican" in the instructions were randomized.

> On each of the next two pages, you will see a list of words. For each word, imagine you were watching a congressional debate on C-Span and you heard that word. You will be asked to guess whether the word you heard was spoken by a Democrat or by a Republican [Republican or by a Democrat]. At the end of this survey, you will be asked whether or not you were paying attention. In order for us to know you read these instructions carefully, please include the word 'ATTENTION' in all caps in your answer to this question.
>
> Your responses will be most helpful if you go with your first impression, so we ask that you spend no more than 5 seconds on each word.

B.3.2 Stimuli

Democratic word	Republican word	Democratic word	Republican word
comprehensive	complete	kinds	types**
wealth	prosperity	end	finish
workers	employees**	criminal	illegal
contribute	give	spoke	talked
values	principles	changes	reforms
assault	attack	mother	mom
acknowledge	recognize	responsibility	duty
fear*	terror*	barriers	walls
respond	answer	indicated	announced
profit	gain**	outrageous	excessive
potentially	possibly	discussion	conversation
glad	happy	maintain**	preserve
financial	monetary	basic	fundamental**

* Excluded from analysis based on not meeting the criteria of substitutability laid out in S3 Appendix.

** Not significantly different from 0 at the 95% level. Confidence intervals are non-parametrically bootstrapped. Simulated corpora are created by randomly resampling with replacement at the speech level from the Congressional Record corpus.

B.4 Study 3b materials

B.4.1 Task instructions for Study 3b

The order of the words "Democrat" and "Republican" in the instructions were randomized.

> On the next few pages, you will see lists of words. For each list of words, imagine you were watching a congressional debate on C-Span and you heard those words. You will be asked to guess whether the words you heard were spoken by a Democrat or by a Republican [Republican or by a Democrat]. At the end of this survey, you will be asked whether or not you were paying attention. In order for us to know you read these instructions carefully, please include the word 'ATTENTION' in all caps in your answer to this question.
>
> Your responses will be most helpful if you go with your first impression, so we ask that you spend no more than 5 seconds on each list.

B.4.2 Task instructions for valence ratings

These task instructions were adapted as closely as possible from Warriner, Kuperman, and Brysbaert (2013) [2]. Also following Warriner et al. (2013) [2], we instructed participants that a rating of "1" corresponded to "happy" and that a rating of "9" corresponded to "unhappy," and reverse-coded our data (so that a higher rating corresponded to a more positively-valenced reaction). The analyses we report that use valence data always shift this data so the midpoint falls at 0, with lower and upper bounds at -4 and 4, respectively.

> You are invited to take part in the study that is investigating emotion, and concerns how people respond to different types of words. You will use a scale to rate how you felt while reading each word. There will be 25 words. The scale ranges from 1 (happy) to 9 (unhappy).
>
> At one extreme of this scale, you are happy, pleased, satisfied, contented, hopeful. When you feel completely happy you should indicate this by choosing rating 1. The other end of the scale is when you feel completely unhappy, annoyed, unsatisfied, melancholic, despaired, or bored. You can indicate feeling completely unhappy by selecting 9. The numbers also allow you to describe intermediate feelings of pleasure, by selecting any of the other feelings. If you feel completely neutral, neither happy nor sad, select the middle of the scale (rating 5). At the end of this survey, you will be asked whether or not you were paying attention. In order for us to know you read these instructions carefully, please include the word 'ATTENTION' in all caps in your answer to this question.
>
> Please work at a rapid pace and don't spend too much time thinking about each word. Rather, make your ratings based on your first and immediate reaction as you read each word.

References

1. Sloman SJ, Oppenheimer D, DeDeo S. One Fee, Two Fees; Red Fee, Blue Fee: People Use the Valence of Others' Speech in Social Relational Judgments; under review.
2. Warriner AB, Kuperman V, Brysbaert M. Norms of Valence, Arousal, and Dominance for 13,915 English Lemmas. Behavior Research Methods. 2013;45(4):1191–1207. doi:10.3758/s13428-012-0314-x.
