# Supplementary material for: Can we detect conditioned variation in political speech? two kinds of discussion and types of conversation
Source: PLoS One. 2021 Feb 11;16(2):e0246689. doi: 10.1371/journal.pone.0246689 (PMC7877629; doi:10.1371/journal.pone.0246689)
Supplement: S3 Appendix — (PDF) [file pone.0246689.s006.pdf]

C Substitutability ratings for items in Study 3

- For each word, we sampled 10 sentences from the Congressional Record that contain that word, and generated 10 new sentences where that word is substituted by its *sense* equivalent.
- We then divided the 1,040 ($26 \times 2 \times 10 \times 2$) sentences into two groups of 520 sentences, ensuring the original and substitution version of a sentence did not appear in the same group.
- We gave four annotators (two per group) the following instructions:

  > The csv file you've been emailed contains a list of 520 sentences. All of these sentences, or versions that are almost identical, were said on the floor of the U.S. House of Congress between 2012 and 2017.
  >
  > For your task, assume that the scribe who has created the transcripts you are reading has made a few mistakes. For example, they may have accidentally transcribed the wrong word here and there. In the sentences that follow, please indicate your judgment of **the likelihood that such an error has been made**.
  >
  > In the "ratings" column, please put a number between 1 and 4, where
  >
  > – 1 means "I am almost sure this is the original text",
  >
  > – 2 means "I am not sure, but this is probably the original text",
  >
  > – 3 means "I am not sure, but this sentence probably contains at least one transcription error", and
  >
  > – 4 means "I am almost sure this sentence contains at least one transcription error".

- For each item (word pair), we pre-committed to excluding it if
  - The correlation between the ratings of members of either group on sentences corresponding to that item was not greater than 0, or
  - If the mean rating of the substitution sentence was higher than the mean rating of the original sentence (the substitution sentence was judged as more likely to have been a transcription error) for at least 13 out of the 20 sentences pertaining to the item.
