# Supplementary material for: Can we detect conditioned variation in political speech? two kinds of discussion and types of conversation
Source: PLoS One. 2021 Feb 11;16(2):e0246689. doi: 10.1371/journal.pone.0246689 (PMC7877629; doi:10.1371/journal.pone.0246689)
Supplement: S4 Appendix — (PDF) [file pone.0246689.s007.pdf]

D Regression results for Study 3

D.1 Study 3a

We pre-registered the following specification for a logistic regression model:

$$log\frac{P(REPUBLICAN)}{1-P(REPUBLICAN)} = \alpha_i + \beta_0 + \beta_1 is\_republican(w) + \beta_2 valence(w) + \beta_3 party(participant) + \beta_4 valence(w) \times party(participant) \quad (2)$$

The model included an L1 penalty, which is imposed to encourage sparsity among the model parameters and is often used for feature selection [1, 2]. α_i indicates the inclusion of participant fixed effects (the inclusion of item-level effects does not affect our conclusions).

The unit of observation in this model is each response ($n = 2,498$). Responses were binarized such that a response of 1, 2 or 3 corresponded to $REPUBLICAN = 0$, while a response of 4, 5 or 6 corresponded to $REPUBLICAN = 1$. $is\_republican(w)$ is 1 if the word being responded to is Republican, and 0 otherwise. $valence(w)$ is the mean valence rating of w in a database collected and made available by Warriner et al. (2013) which contains ratings of the perceived valence of thousands of English words [3]. $party(participant)$ is 1 if the participant responding self-identifies as a Republican, and -1 if they self-identify as a Democrat (this model excluded responses by the 52 participants who did not self-identify as a Democrat or a Republican). We also excluded responses to nine of the items for which we didn't have valence data (four Democratic items and five Republican items).

The estimated coefficients are shown in Table 1. Table 1 also shows the estimated coefficients in an L1-penalized linear regression model in which the raw ratings data is used as the dependent variable. To check the assumptions of linearity made by the regression models reported in this section, we also estimated generalized additive models with the same specifications. Because these models suggest that these relationships are not linear, we do not interpret the magnitude of our coefficients.

Table 1. Estimated coefficients in the regression models.[a]

DV	Binary		Ordinal	
$is\_republican(w)$	.11		.23	
$logodds_R(w)$		.24		.23
$valence(w)$	-.12	-.11	-.12	-.11
$party\_id(participant)$	.65	.65	.18	.20
$valence(w) \times party\_id(participant)$	.09	.09	.12	.12
ROC AUC	.65	.65		
**Adjusted R^2**			.08	.08

[a] Binary DV indicates that the ratings scale was binarized, and the estimation procedure was an L1-penalized logistic regression. Ordinal DV indicates that observations were the ratings on the six-point scale, and the estimation procedure was an L1-penalized linear regression.

The models described above regress against a binary indicator that is 1 if the stimulus is Republican, and 0 if the stimulus is Democratic. We also ran versions of these models that included the continuous $logodds_R$ values (positive if the word is more likely to have been spoken by a Republican, and negative if the word is more likely to have been spoken by a Democrat). Table 1 also shows the results of these regressions. The coefficients in the row labelled $logodds_R$ are positive, indicating that at a given level of *valence*, *party* and $valence \times party$, higher politically conditioned variation is correlated with a higher subjective likelihood that the word is spoken by a Republican.

It is worth noting that the coefficients on the valence bias terms $valence \times party$ are consistently positive, which is further indicative of the persistence of the social valence bias.

D.2 Study 3b

Table 2 shows the estimated parameters of regression models with the specification given in Eq. 2.

To accommodate differences in the task format, we made slight changes to how we operationalized some of the independent variables listed in Table 2. In particular, we considered the relative frequency terms to be the sum of the $logodds_R$ values of each word in the list, and the valence terms to be the sum of the valences of each word in the list.

The Warriner et al. (2013) database [3] used to operationalize valence in the model

Table 2. Estimated coefficients in the regression models of the data from Study 3b.[b]

DV	Binary		Ordinal	
$is\_republican(w_{i \in 1:5})$	.77		.61	
$\sum_i logodds_R(w_i)$		.18		.14
$\sum_i valence(w_i)$	-.07	-.06	-.06	-.05
$party(participant)$	-.10	-.10	-.11	-.09
$\sum_i valence(w_i) \times party\_id(participant)$	.06	.06	.03	.03
ROC AUC	.82	.81		
**Adjusted R^2**			.02	.02

[b] Binary DV indicates that the ratings scale was binarized, and the estimation procedure was an L1-penalized logistic regression. Ordinal DV indicates that observations were the ratings on the six-point scale, and the estimation procedure was an L1-penalized linear regression.

estimated on the data from Study 3a is missing data for nine of the items we used in Studies 3a and 3b. In a study whose instructions and response options were designed to closely resemble the study run by Warriner et al. (2013) [3], we collected valence ratings on the 50 words. The exact wording of the instructions given to participants is included in S2 Appendix. The randomization was the same as in Study 3a. 41 participants completed this study on MTurk, and 15 were excluded on the basis of failing the instructional manipulation check. In the analysis reported in this section, the valence of a word is operationalized as the mean rating of a word from these data.

References

1. Santosa F, Symes WW. Linear Inversion of Band-Limited Reflection Seismograms. SIAM Journal on Scientific and Statistical Computing. 1986;7(4):1307–1330. doi:10.1137/0907087.

2. Tibshirani R. Regression Shrinkage and Selection Via the Lasso. Journal of the Royal Statistical Society: Series B (Methodological). 1996;58(1):267–288. doi:10.1111/j.2517-6161.1996.tb02080.x.

3. Warriner AB, Kuperman V, Brysbaert M. Norms of Valence, Arousal, and Dominance for 13,915 English Lemmas. Behavior Research Methods. 2013;45(4):1191–1207. doi:10.3758/s13428-012-0314-x.
