# Supplementary material for: Can we detect conditioned variation in political speech? two kinds of discussion and types of conversation
Source: PLoS One. 2021 Feb 11;16(2):e0246689. doi: 10.1371/journal.pone.0246689 (PMC7877629; doi:10.1371/journal.pone.0246689)
Supplement: S5 Appendix — (PDF) [file pone.0246689.s008.pdf]

E Analysis of hypothesized mediators

In Study 1, we found a statistically significant difference in the discriminabilities (defined in our discussion of Study 1) of self-identified Democrats and Republicans ($\mu_D = .47$; $SE_D = .04$; $\mu_R = .24$; $SE_R = .06$; $t_{65.57} = 3.41$; $p < .01$). In this section, we report the results of an analysis that controlled for other hypothesized mediators of discriminability that may interact with a participant's party affiliation.

We wondered if the importance of the dimension of political identity to a participant would predict their sensitivity to politically conditioned variation, suggesting a "top-down" mechanism for higher discriminability. On the other hand, high discriminability could reflect a participant's exposure to political speech. If a person is more exposed to the ecological distribution of political speech, they may be better able to use these "bottom-up" inputs to reconstruct these distributions.

In addition, the social valence bias discussed in Sloman et al. (under review) [1] could skew the distributions of responses. Since we did not attempt to balance the valence of the Republican and Democratic items, this effect could result in asymmetries in the accuracy of judgments that are due to a property of the stimulus set, not a difference in the capacity for signal recovery in the two groups.

We used participant responses on our demographic questionnaires as proxies for the strength of their partisan identity and degree of exposure to political speech. Participants were asked to respond to the question "How strongly do you identify with your party?" on a 1 to 7 scale (where a response of 1 indicated "very little" and a response of 7 indicated "very much"). We use this as a proxy of the strength of their partisan identity. We also asked participants "How engaged are you in politics?" on the same scale. We use this as a proxy of their degree of exposure to political speech, under the assumption that participants who are more engaged in politics would tend to be exposed to more public-facing political speeches and media.

To test if the relationship between party affiliation and discriminability persisted when we controlled for these other factors, we estimated a logistic regression model that regressed a binary variable indicating the correctness of each response against these other factors (see Dixon (2008) for an argument to use logistic regression models to analyze accuracy data [2]). We consider a response to be "correct" if the participant judges a word w as more likely to have been spoken by a Democrat (a judgment of 1, 2 or 3) and $logodds_R(w) < 0$, or the participant judges the word as more likely to have been spoken by a Republican (a judgment of 4, 5 or 6) and $logodds_R(w) > 0$.

We estimated the parameters of the following model:

$$\begin{aligned} log\frac{P(CORRECT)}{1-P(CORRECT)} = &\beta_0 + \beta_1 logodds(w) + \beta_2 log(P(w)) \\ &+ \beta_3 party(participant) + \beta_4 valence(w) \\ &+ \beta_5 party(participant) \times valence(w) \times is\_republican(w) \\ &+ \beta_6 strength\_party\_id(participant) \\ &+ \beta_7 party(participant) \times strength\_party\_id(participant) \\ &+ \beta_8 exposure(participant) \\ &+ \beta_9 party(participant) \times exposure(participant) \end{aligned} \tag{3}$$

The model included an L1 penalty. We did not include participant- or item-level fixed effects because doing so introduced multicollinearities.

The unit of observation in this model is each response by a participant who self-identified as a Democrat or a Republican ($n = 6,726$). $logodds(w)$ is the absolute value of the $logodds_R$ of the item w (which is equivalent to the absolute value of the $logodds_D$ of the same item). $log(P(w))$ is the log of the marginal probability of the word occurring in the Congressional Record. It was included to control for any effect the familiarity of the word contributed to participants' ability to encode the politically conditioned variation (see Shah and Oppenheimer (2008) for the effect of fluency on human judgment and decisions [3]). *party*(*participant*) is 1 if the participant responding self-identifies as a Republican, and -1 if they self-identify as a Democrat. *valence* is the mean valence rating of a word in the data collected by Warriner et al. (2013) [4]. *is republican* takes on a value of 1 if w is Republican ($logodds_R(w) > 0$) and -1 if w is Democratic ($logodds_R(w) < 0$). The $party \times valence \times is\_republican$ term directly captures the effect of the social valence bias: It is positive in cases where w is positively-valenced and the direction of the *logodds* term corresponds with *party*, and in cases where w is negative and the direction of *logodds doesn't* correspond with *party*. Conversely, it is negative in cases where w is positive and the direction of *logodds* does not correspond with *party*, and in cases where w is negative and *logodds* does correspond with *party*. Responses to the strength of party identity and political engagement questions mentioned above are included as *strength_party_id* and *exposure*,

Table 3. Estimated parameters of the model in Eq. 3.

	Study 1	Study 2	Study 3a	Study 3b
logodds	.33	.17	.09	
$log(P)$	.13	.07	-.33	-.16
party	-.08	.04	.07	
valence	.19	-.03	-.19	.03
party × *valence* × *is_republican*	.30	.34	.39	
strength_party_id	.02	-.03	.02	
party × *strength_party_id*	-.02	-.03	-.02	
exposure	.06	.08	.06	
party × *exposure*	.01	.02	-.01	
ROC AUC	.61	.60	.65	.60

respectively. Both are rescaled so 0 corresponds to the midpoint of the scale.

The $log(P(w))$ term tended to have a larger absolute value than the other terms. We thought this might result in the suppression of the effects of other variables, and therefore standardized all the variables. (In other regression models reported in this paper, we use the raw (unstandardized) data.) We excluded responses to seven of the items for which we didn't have valence data.

Table 3 shows the estimated coefficients of this model. When these other factors are controlled for, this model estimates an extremely small effect of a person's political affiliation on their accuracy.

For completeness, we estimated the same model on the data from Studies 2 and 3. (We didn't find a statistically significant difference in performance between Republicans and Democrats in any of these studies.) We slightly modified the specification of terms of the model to accommodate the different task formats. For Study 2, the *logodds* term equals the sum of the *logodds* values of the words in the pair, the $log(P(w))$ term equals the sum of the $log(P(w))$ values of the words in the pair, and *valence* equals the difference in valence scores between the word the participant chose and the word they didn't choose, and *is_republican* takes on 1 if the word they chose is Republican, and -1 if the word they chose is Democratic. For Study 3b, the *logodds* term equals the sum of the $logodds_R$ values of each word in the list, the $log(P(w))$ term equals the sum of the $log(P(w))$ values of each word in the list, and *valence* equals the sum of the average valence ratings for each word in the list. In all three additional models, the coefficient on the *party* term is even smaller in magnitude, and in no model is the direction consistent with the effect suggested by the data from Study 1. We therefore conclude that there is

no reliable effect of a participant's political affiliation on their discriminability.

References

1. Sloman SJ, Oppenheimer D, DeDeo S. One Fee, Two Fees; Red Fee, Blue Fee: People Use the Valence of Others' Speech in Social Relational Judgments; under review.
2. Dixon P. Models of Accuracy in Repeated-Measures Designs. Journal of Memory and Language. 2008;59(4):447–456. doi:10.1016/j.jml.2007.11.004.
3. Shah AK, Oppenheimer DM. Heuristics Made Easy: An Effort-Reduction Framework. Psychological Bulletin. 2008;134(2):207–222. doi:10.1037/0033-2909.134.2.207.
4. Warriner AB, Kuperman V, Brysbaert M. Norms of Valence, Arousal, and Dominance for 13,915 English Lemmas. Behavior Research Methods. 2013;45(4):1191–1207. doi:10.3758/s13428-012-0314-x.
