# Supplementary material for: Can we detect conditioned variation in political speech? two kinds of discussion and types of conversation
Source: PLoS One. 2021 Feb 11;16(2):e0246689. doi: 10.1371/journal.pone.0246689 (PMC7877629; doi:10.1371/journal.pone.0246689)
Supplement: Attachment — Submitted filename: Response to Reviewers.docx [file pone.0246689.s009.docx]

Dear Dr. Thomas Holtgraves,

Thank you for your careful consideration of and helpful feedback on our manuscript “Can we detect conditioned variation in political speech? Two kinds of discussion and types of conversation.” Our revised manuscript incorporates all the feedback provided by you and the two reviewers. Below, we respond to each point raised by you and the reviewers.

1. *First, your main argument hinges on the distinction between semantic and statistical information.  As reviewer 2 notes, however, that distinction is not clear-cut and some approaches equate the two. So this needs to be clarified and I think editing and changes in terminology (as suggested by Reviewer 2) is probably the way to go, although there may be other possibilities.*

Based on these comments, we have revised our terminology for distinguishing between statistical and semantic information. As Reviewer 2 rightly points out, the concept of semantic information is not well-defined, and our original terminology did not make it clear how it was distinct from statistical information. We hope our choice of the phrases “politically conditioned variation” (what our original manuscript referred to as “statistical information;” defined on lines 50--52) and “word *sense*” (what our original manuscript referred to as “semantic information;” defined on lines 28—29) make this distinction clearer.

2. *Second, the description of the methods and the reporting of the results requires a major overall.  In many places it is difficult to tell how the analyses were conducted and in other places it is unclear what the results mean.  For example when you say mixed model you should provide more information regarding the nature of that model (e.g., what were the random and fixed effects?). And for all reported effects you should follow APA guidelines and report the df and effect sizes. Reviewer 2 has multiple, detailed comments in this regard and you should respond to all of them.*

As you and Reviewer 2 correctly point out, in our original manuscript we did not follow a standardized format for our results sections, did not break up our hypothesis tests by the partisan polarity of the stimuli, and did not report standardized effect sizes. Following helpful suggestions from Reviewer 2, we have broken up each of our results sections into subsections that detail our main analyses, participant-level analyses and item-level analyses, respectively. In the main analysis sections, we report a standardized effect size for each study, using the method of Rouder, Morey, Speckman, and Province (2012). Our reporting of the results of Studies 1 and 3 include the same analyses reported in the same order. While the design of Study 2 differs slightly, we follow the same analysis strategy and reporting format to the extent possible.

In addition to standardizing our analysis strategies and formatting, we have substantially revised our results sections in other ways:

- Based on Reviewer 2’s comments, we also include extensive analysis and discussion of differences in results when only Republican [Democratic] items are considered.
- Based on Reviewer 2’s comments, we have included new figures that draw on what was Fig 3 in the original submission as inspiration.
- We have clustered standard errors used for our main analyses at the participant- and item-level.
- The tables in Appendices D and E contain slightly modified coefficient estimates that reflect minor changes to our model estimation strategies (using an L1 penalty in favor of OLS for the linear regressions in Appendix D and the inclusion of item-level effects in Appendix E). Most notably, in our original manuscript, we mentioned that a pre-registered regression model of the data from Study 3a (first column in Table 1) did not estimate a coefficient on *logodds_R_* in the hypothesized direction. This was due to a programming error; when this is fixed, this model *does* show a coefficient estimate in the hypothesized direction. (None of the other changes affect our central theoretical claims.)

3. *Third, both reviewers encourage greater contextualization of your work and encourage additional speculation regarding the meaning of your findings, especially in the context of single words vs. phrases and sentences. Although this is less critical, these comments do need to be addressed.*

There are a number of places where we have clarified our methods and included additional points of discussion based on feedback from both reviewers:

- On lines 790--797, we clarify our rationale for our choice to investigate politically conditioned variation at the level of single words rather than phrases or sentences. To summarize, this choice was in our judgment necessary to adequately control for differences in word *sense* between our stimuli.
- Both reviewers recommended we use a different example to highlight the distinction between politically conditioned variation and word *sense*. We have replaced the example of “gun control” vs. “gun laws” with “financial” (a Democratic word) vs. “monetary” (a Republican word; lines 27--44). We believe this addresses the concern raised by Reviewer 1 that the example included bigrams while our analyses occurred at the level of unigrams, and that our discussion on lines 27--34 addresses the concern raised by both reviewers that the example did not make salient our notion of semantic equivalence (i.e. *sense* equivalence).
- In response to Reviewer’s 2 request that we speculate on the downstream consequences of sensitivity to politically conditioned variation, we include an additional section in our discussion entitled “Consequences of sensitivity to politically conditioned variation” (lines 904--926). Reviewer 2 noted that our findings could have important implications for political persuasion and the spread of fake news. We elaborate on this, providing additional discussion about how detection of politically conditioned variation may allow people to make additional inferences about the speaker, and, especially to the extent that it provides signals to ingroup or outgroup membership, could lead to differences in perceived trustworthiness and credibility.
- In lines 868—872, we include Reviewer 1’s insightful point that there are likely latent factors that reflect other differences between Republicans and Democrats, yet manifest themselves as politically conditioned variation.
- Reviewer 1 also notes that we did not justify our decision to use data from a five-year window of time to measure politically conditioned variation. We explain our rationale for this choice on lines 122--130. To summarize, we believe this time window achieves a balance between collecting enough data to recover reliable trends and our concern that a time window that extended back too far would overwhelm trends from more recent years.

Reviewer 2 also provided a number of additional line-by-line comments, each of which has been addressed in the revised manuscript:

1. We have altered the sentence in the abstract from “In a series of four studies, we demonstrate that participants’ judgments **align with the direction of statistical information** more often than chance, and that this effect persists even when potentially interfering cues such as the **semantic content of the words** are controlled for” to “In a series of four studies, we demonstrate that participants’ judgments **track variation in word usage between the two parties** more often than chance, and that this effect persists even when potentially interfering cues such as the **meaning of the word** are controlled for.”
2. On lines 63--75, we spell out the practical relevance of our work. To summarize, understanding sensitivity to politically conditioned variation can help us better understand the kinds and validity of cues we use to make implicit judgments about others.
3. We have attempted to clarify that Reviewer 2’s interpretation that classifications of words are based on the sign of their *logodds_R_* value is correct by altering our phrasing on lines 162--163 to stress that classification depends on *logodds_R_* being greater than or less than 0. Reviewer 2 also recommends we report and comment on the distribution of *logodds_R_* values in the Congressional Record corpus. We have replaced Fig 1 (restating the important takeaways from this figure in the text; lines 188--196) with a figure visualizing this distribution. In addition, we address Reviewer 2’s question of whether this distribution was distinct from a plausible null distribution on lines 164--168.
4. As Reviewer 2 suggests, we re-structure the section “Measures of relative frequency” to focus on the *logodds*_R_ metric. We move discussion of PKL to lines 227--237, and clarify that “PKL” is an abbreviation for “partial Kullback-Leibler divergence.”
5. On lines 188 and 191, we report proportions as well as the absolute number of words that retain their polarity from the Congressional Record to the presidential debates corpora.
6. We provide additional motivation for Study 2 before the corresponding “Participants” and “Methods” sections (lines 338--359).
7. We clarify that stimulus selection for Study 2 entailed 662,596 pairwise comparisons (line 373).
8. We remove the word “strongly” due to Reviewer 2’s observation that “strong rejection” cannot occur in a frequentist statistical framework.
9. Reviewer 2 suggests that the lack of a significant result for the item-level analysis of Study 2 (line 434) was likely due to a lack of power (since there were only 87 items). In the corresponding “Item-level analyses” section, we discuss additional analyses showing that performance *was* significantly higher than chance for participants in one of the two conditions. In light of other results from Study 2, we chose to stress the difference between conditions in discussion of the item-level result. However, we are happy to also speculate about the power of our tests if the reviewer believes it would aid readers’ interpretation of our results.
10. We have included degrees of freedom in reporting of all of our *t-*tests. While we believe Cohen’s *d*s are difficult to interpret for repeated measures data (see our discussion on lines 268--282), we provide a repeated measures analog developed by Rouder et al. (2012; see discussion above).
11. As mentioned above, we clarify our rationale for focusing on word-level effects on lines 782--794.
12. As mentioned above, we speculate on the consequences of sensitivity to politically conditioned variation in the section “Consequences of sensitivity to politically conditioned variation.”

Finally, we would like to respond here to your questions about overlap between the manuscript we have submitted to your journal (the present manuscript) and another manuscript of ours that is currently under review entitled “One Fee, Two Fees; Red Fee, Blue Fee: People Use the Valence of Others’ Speech in Social Relational Judgments.” The present manuscript addresses the research question “Can participants detect politically conditioned variation?” Sloman, Oppenheimer, and DeDeo (under review) addresses the research question “Do participants perceive the speech of members of their political in-group as more positive than the speech of members of their political out-group?” Addressing both research questions required analysis of participant judgments of the most likely political identity of a hypothetical speaker. We therefore found it convenient to ask participants in Studies 1 and 2 of the present manuscript to also make judgments about words that would test the research question addressed in Sloman et al. (under review). In Appendix B of the present manuscript, you and interested readers can see the full list of items participants in Studies 1 and 2 were presented with. We have clearly demarcated which of these items were included to test the research question addressed in the present manuscript, and which of these items were included to test the research question addressed in Sloman et al. (under review). These lists do not overlap; in other words, none of the data analyzed for and reported in the present manuscript is reported in Sloman et al. (under review). Since a) the research questions addressed in the present manuscript and in Sloman et al. (under review) are completely distinct, and b) none of the data analyzed for or reported in the present manuscript is reported in Sloman et al. (under review), we believe this **does not** constitute dual publication.

We hope our revisions adequately address your concerns about the clarity of our theoretical and methodological approach. We thank you and the reviewers again for the time taken to provide feedback on our work, and look forwards to your replies.

Sincerely,

Sabina J. Sloman

Dr. Daniel Oppenheimer

Dr. Simon DeDeo

**References**

Rouder, J.N., Morey, R.D., Speckman, P.L., & Province, J.M. (2012). Default Bayes factors for ANOVA designs. *Journal of Mathematical Psychology, 56*(5), 356-374.

Jordan, K.N. & Pennebaker, J.W. (2017). The Exception or the Rule: Using Words to Assess Analytic Thinking, Donald Trump, and the American Presidency. *Translational Issues in Psychological Science, 3*(3), 312-316.

Sloman, S.J., Oppenheimer, D., & DeDeo, S. (under review). One Fee, Two Fees; Red Fee, Blue Fee: People Use the Valence of Others’ Speech in Social Relational Judgments.
