# Supplementary material for: Can we detect conditioned variation in political speech? two kinds of discussion and types of conversation
Source: PLoS One. 2021 Feb 11;16(2):e0246689. doi: 10.1371/journal.pone.0246689 (PMC7877629; doi:10.1371/journal.pone.0246689)
Supplement: Attachment — Submitted filename: Response to Reviewers.docx [file pone.0246689.s010.docx]

Dear Dr. Thomas Holtgraves,

Thank you for your feedback on our manuscript “Can we detect conditioned variation in political speech? Two kinds of discussion and types of conversation.” Below, we point to where in our manuscript we have addressed each point you raised.

1. *First, please address all of the minor issues raised by Reviewer 2.*

The reviewer’s comments were (lettered rather than numbered to avoid confusion with your comments):

a. *I might have missed this the first time, but I was surprised by the fact that the correlation between log-odds for Congressional and Presidential speeches was only a small to medium correlation. Although it is significant (which can provide some validation of the method), it also points to the variation in Congressional vs. Presidential speeches. A single sentence or two elaborating on these differences would be helpful, either in the main text or SM. Where is this variation coming from?*

We agree with the reviewer’s insight that there is still much variation to be explained between the two corpora. While we cannot completely explain the sources of divergence, we include an additional supplementary figure, S1 Fig, that shows how the generalizability of politically conditioned variation varies with its strength in the Congressional Record corpus. We find that the strongest political signals are also the most generalizable. We summarize these findings on lines 197—202.

b. *Relatedly, which corpus is actually better reflection of the signals that lay perceivers are learning from? Presumably the Presidential speeches, as the authors themselves note. So why did the authors not use that as the primary corpus of interest for the authors initial generation of word stimuli? I understand the points the authors made to justify using the Congressional speeches, but if their goal is to look at lay perception of politically conditioned variation, then doesn’t it make sense to look at the most “lay” corpus for word differences? I also realize it’s impossible to go back and change this now, but it might help to address this in a footnote as a limitation or direction for future work.*

We address this limitation on lines 875—885 of our general discussion.

c. *Line 260 “to reject the null that this value was greater than or equal to…” – is this inclusion of “greater than” correct? It seems like you rejected the null that the value was equal to the indifference point. The same issue for the Democratic stimuli reported in the following paragraph. Please double check or clarify.*

The reviewer is correct to point out that this was a typo on our part. We have adjusted the sentence to read “A one-sided, one-sample t-test led us to reject the null that this value was less than or equal to the indifference point…” (lines 282—284). We hope this clarifies that we performed a one-sided test to test our alternative hypothesis that participants’ judgments on the Republican items would fall *above* the indifference point. We have also adjusted terminology when reporting results on the Democratic items (lines 289—290).

d. *Thank you for the additional clarity about the mixed model and effect size estimation.*
e. *Line 373 – where did the choice of N = 88 pairs come from? Was there some principled cut-off for cosine similarities? Would choosing only the highest cosine similarity pairs weaken the effect even further? Such a finding would suggest that differences in word sense do, in fact, have some impact on the discrimination of R vs. D words.*

On lines 401—406, we clarify that we predetermined that we would present participants with 100 items, ten of which were included to test the hypothesis discussed in Sloman, Oppenheimer, and DeDeo (under review) and two of which were included as part of our attention check. In other words, the choice of 88 pairs was an artifact of experimental constraints, not the result of a constraint imposed by the distribution of cosine similarities.

f. *Another alternative for why Republican discriminability may be higher (in addition to the frequency of exposure to speech as the authors mentioned) is that lay perceivers may have stronger stereotypes about Republicans than about Democrats. In other words, they may have clearer notions about the traits and cognitions of Republicans than Democrats. My intuition is that, especially during Trump’s presidency, lay perceivers see Republicans as a more extreme social group than Democrats. Another speculation to play with, perhaps?*

We interpret the reviewer’s comment as suggesting that participants’ cognitive representations of Republican speech were stronger and easier to access. We include this suggestion on lines 689—692, and believe it also links with the section of our general discussion on lines 838—854.

g. *The figures are all much improved!*

2. *Second, because your samples consist of  MTurk workers I would like you to mention some of the limitations (e.g., non-naivety and trustworthiness) of using participants from this platform.*

   On lines 215—229, we provide a brief discussion of the advantages and limitations of using MTurk as a recruiting tool, in addition to discussion of how these limitations would affect interpretation of our results.
3. *Third, for study 2, state in the method section that participants provided judgments of 100 word pairs, 12 of which were for a different study.*

   As mentioned in our response to 1e, we clarify on lines 401--406 that we predetermined that we would present participants with 100 items, ten of which were included to test the hypothesis discussed in Sloman et al. (under review) and two of which were included as part of our attention check.
4. *Fourth, please add a sentence within the manuscript that provides a reference to the location of your data and code (and please make sure that the studies in this manuscript are aligned with what is contained on your GitHub page).*

   We include the link to the GitHub repository on line 277. We have also updated the repository to align with the analyses and results reported in the submitted version of the manuscript.

We hope our revisions adequately address your concerns. We thank you and the reviewer for the time taken to provide feedback on our work.

Sincerely,

Sabina J. Sloman

Dr. Daniel Oppenheimer

Dr. Simon DeDeo

References

Sloman, S.J., Oppenheimer, D., & DeDeo, S. (under review). One Fee, Two Fees; Red Fee, Blue Fee: People Use the Valence of Others’ Speech in Social Relational Judgments.
